# Species composition of small mammal communities and prevalence of *Schistosoma japonicum* in the Poyang Lake region, China

**DOI:** 10.1051/parasite/2026051

**Published:** 2026-09-14

**Authors:** Xiaodong Weng, Mingxiao Di, Bo Zhou, Chen Zhang, Jiaxin Zheng, Ping Lu

**Affiliations:** 1 Institute of Resources and Environment, Jiangxi Academy of Sciences Nanchang 330096 PR China; 2 School of Life Sciences, East China Normal University Shanghai 200241 PR China

**Keywords:** Small mammals, *Schistosoma japonicum*, Poyang Lake region, *Apodemus agrarius*, *Rattus losea*

## Abstract

Small mammals are an important part of the ecosystem and can serve as the definitive host of *Schistosoma japonicum*. The Poyang Lake region of Jiangxi Province is one of the most important endemic areas of schistosomiasis japonica in China. However, data on the prevalence of schistosomiasis in local small mammal communities are lacking. Therefore, from 2021 to 2022, we captured 409 small mammals in four townships in this region, with a total capture rate of 6.89% (409/5938). There were significant differences in the capture rate among different habitats (*χ*^2^ = 100.74, *p* < 0.001) and different sampling sites (*χ*^2^ = 100.74, *p* < 0.001), but there was no significant difference between sampling seasons (*χ*^2^ = 4.437, *p* = 0.109). Traditional morphology combined with molecular DNA-barcoding was effective in identifying species, and four species, *Apodemus agrarius, Rattus losea, R. norvegicus* and *Crocidura attenuata* were identified. PCR of the *Sjr2* gene showed a total prevalence of *S. japonicum* of 2.85% (8/281) in *A. agrarius* and 2.83% (3/106) in *R. losea*. The species, sex, age, sampling sites, seasons, and habitats were not significant factors influencing the prevalence of *S. japonicum* in small mammals. This study revealed the molecular prevalence of *S. japonicum* in the wild small mammal community in the Poyang Lake region for the first time. Monitoring schistosomiasis prevalence in *A. agrarius* and *R. losea* may contribute to future elimination efforts around Poyang Lake.

## Abstract (fr)

Les petits mammifères constituent une composante importante de l'écosystème et peuvent servir d'hôtes définitifs pour *Schistosoma japonicum*. La région du lac Poyang, dans la province du Jiangxi, est l'une des principales zones d'endémie de la bilharziose à *S. japonicum* en Chine. Toutefois, les données relatives à la prévalence de cette maladie au sein des communautés locales de petits mammifères font défaut. Ainsi, entre 2021 et 2022, nous avons capturé 409 petits mammifères dans quatre communes de cette région, soit un taux de capture global de 6,89 % (409/5938). Des différences significatives ont été observées concernant le taux de capture selon les habitats (χ^2^ = 100,74 ; *P* < 0,001) et les sites d'échantillonnage (χ^2^ = 100,74 ; *P* < 0,001), mais aucune différence significative n'a été relevée entre les saisons d'échantillonnage (χ^2^ = 4,437 ; *P* = 0,109). L'approche combinant morphologie classique et code-barres ADN s'est avérée efficace pour l'identification des espèces. Quatre espèces ont ainsi été identifiées : *Apodemus agrarius*, *Rattus losea*, *R. norvegicus* et *Crocidura attenuata*. L'analyse par PCR du gène *Sjr2* a révélé une prévalence globale de *S. japonicum* de 2,85 % (8/281) chez *A. agrarius* et 2,83 % (3/106) chez *R. losea*. L'espèce, le sexe, l'âge, les sites d'échantillonnage, les saisons et les habitats ne constituaient pas des facteurs influençant de manière significative la prévalence de *S. japonicum* chez les petits mammifères. Cette étude a permis de déterminer, pour la première fois, la prévalence moléculaire de *S. japonicum* au sein des communautés de petits mammifères sauvages de la région du lac Poyang. Le suivi de la prévalence de la bilharziose chez *A. agrarius* et *R. losea* pourrait contribuer aux futurs efforts d'élimination autour du lac Poyang.

## Introduction

Small mammals refer to small and light mammals mainly in the orders Rodentia and Eulipotyphla. They are abundant and widely distributed [19, 56] and can promote energy flow in the ecosystem, assist in the spread of plant seeds, and maintain the stability and biodiversity of the ecosystem [2, 14]. However, they also pose a threat to the production and security of human society. For example, in the middle and lower reaches of the Yangtze River, *Apodemus agrarius*, *Rattus losea*, and *R. norvegicus* have caused huge losses in agricultural production [34, 41]. At the same time, small mammals are hosts of zoonotic pathogens causing diseases such as leptospirosis [3], hemorrhagic fever [21], the plague [44], and others [22, 54, 55]. These diseases can be directly or indirectly transmitted to humans [40]. In recent years, more attention has been paid to the role of small mammals as definitive hosts in the transmission of zoonotic schistosomiasis [23, 43, 52]. Therefore, understanding the ecology of the small mammal community will provide more basic data for the prevention and control of schistosomiasis.

Schistosomiasis is a serious, harmful parasitic disease caused by *Schistosoma* spp*.* infection. The number of individuals infected worldwide was more than 220 million in 2016 [7]. In China, schistosomiasis japonica, caused by *Schistosoma japonicum*, is endemic in the Yangtze River Basin and southern China and was listed as one of the national key parasitic diseases for prevention and control [66]. Poyang Lake is the largest freshwater lake in China, located in the north of Jiangxi Province and in the middle and lower reaches of the Yangtze River. The Poyang Lake region, which includes 11 counties such as Xinjian County, Duchang County and Poyang County, is one of the major endemic areas of schistosomiasis japonica in China [5]. People and livestock (e.g. cattle, domestic pigs) infected with *S. japonicum* are considered the main infection sources in these areas [5]. Following implementation of the comprehensive prevention and control strategy focusing on infection source control in recent years, the infection rate in people (0.004%) and livestock in the Poyang Lake region has been reduced to the lowest level in history [35].

The complete life cycle of *S. japonicum* involves parasitizing *Oncomelania hupensis* as the sole intermediate host and mammals as definitive hosts. Among the more than 40 mammal species known to be naturally infected with *S. japonicum* (belonging to 7 orders and 28 genera), over 20 species are small mammals [27]. Studies have shown that small mammals in the middle and lower reaches of the Yangtze River may have a high prevalence of *S. japonicum*. For example, the prevalence of *S. japonicum* in *R. norvegicus* and *A. agrarius* has reached 61.1% and 16.0% [39], respectively. The total prevalence in wild rodents in mountainous areas of Jiangxi Province could also reach 5.26% [38]. As a group of mammals with the largest number of species and individuals, the strongest adaptability and close relationships with humans, the wide distribution of small mammals provides an ideal way for the hatching and subsequent continuous spread of *S. japonicum* cercariae, and the role of small mammals in the transmission of schistosomiasis japonica cannot be ignored [23, 48]. In order to achieve the goal of comprehensive elimination of schistosomiasis japonica nationwide, determination of the prevalence of *S. japonicum* in wild small mammals and assessing the transmission risk of schistosomiasis japonica are considered to be the key factors requiring more attention [60].

On the one hand, the abundant beaches and lakeside farmland in the Poyang Lake region provide a suitable habitat for small mammals, leading to large populations of these animals [11, 36]. On the other, wild small mammals serve as important definitive hosts for *S. japonicum*, but there is a lack of relevant ecological and epidemiologic information and data reports, urgently necessitating further research in this region. Therefore, we conducted a quarterly survey of small mammals in the Poyang Lake region from 2021 and 2022, using a combination of morphological and molecular biology methods for species identification, followed by the evaluation of *S. japonicum* prevalence in small mammals using polymerase chain reaction (PCR) amplification with specific primers. This study aimed to understand the species composition and population distribution of the small mammal community in the Poyang Lake region and to further determine *S. japonicum* prevalence data among wild small mammals, which is important for the comprehensive elimination of schistosomiasis japonica in this region and in other regions of China.

## Materials and methods

### Ethics

The Animal Care and Ethics Committee of the Institute of Biological Resources, Jiangxi Academy of Sciences approved all protocols of sampling collections (approval No. 2022023).

### Study area

Poyang Lake (28°24′N–29°46′N, 115°49′E–116°46′E) located in the middle and lower reaches of the Yangtze River in Jiangxi Province, is the largest freshwater lake in China. The local climate is mild with abundant rainfall, and a subtropical humid climate. It receives water from five major rivers in Jiangxi Province, namely the Gan, Fu, Xin, Rao, and Xiushui Rivers. After regulation and storage, the water is injected into the Yangtze River through Hukou. It is a transitional, throughput, and seasonal lake. The average annual precipitation in the lake area ranges from 1,387 to 1,795 mm, with significant interannual variation and uneven distribution throughout the year. During the wet season (April to September), the lake area can reach a maximum of 5,156 km^2^, but during the dry season (October to March of the following year), the water surface area is only 556.6 km^2^ [12].

### Sample collection

**Figure 1:**
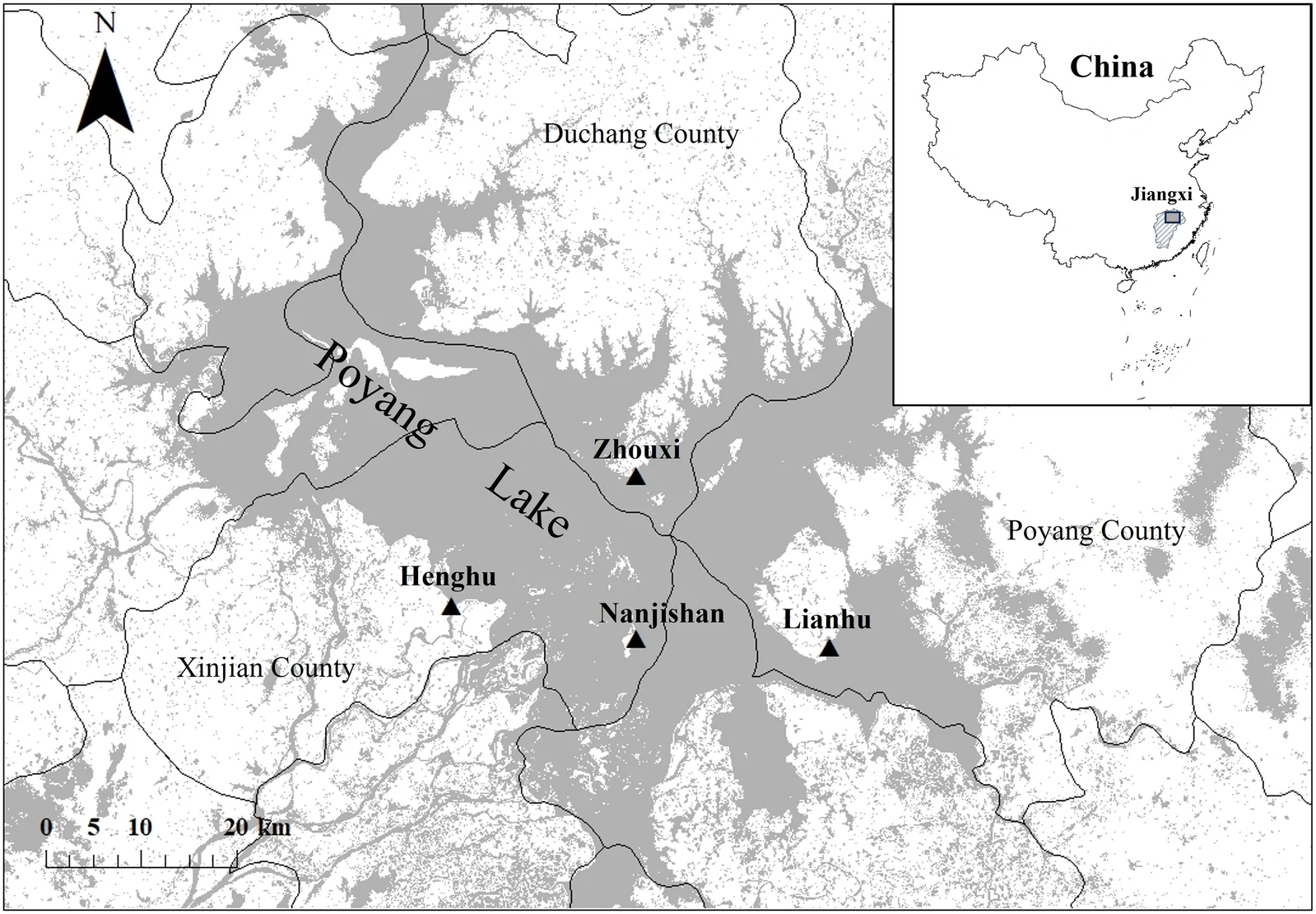
Sampling location in the Poyang Lake region, China.

From September 2021 to June 2022, four sampling locations around Poyang Lake were selected to collect small mammals: Nanjishan (NJS) Township in Xinjian County (28°57′N, 116°21′E), Henghu (HH) Farm in Xinjian County (28°59′N, 116°9′E), Lianhu (LH) Township in Poyang County (28°56′N, 116°34′E), and Zhouxi (ZX) Township in Duchang County (29°6′N, 116°21′E) (Fig. 1). At each sampling site, small mammals were captured using snap traps (150 × 80 mm, Guixi Mousing Tool Factory, Jiangxi, China) on a quarterly basis (September 2021, December 2021, March 2022, and June 2022) in the beach and lakeside farmland habitats. The snap traps were placed at a spacing of 5 m along straight transects. Four transects were set up, with a spacing of more than 150 m between the transects. Each transect contained 70–80 snap traps, with a total of approximately 300 snap traps set up in each habitat. All snap traps were placed at 5:00 pm with fresh sunflower seeds and collected the next day at 8:00 am.

### Species identification of small mammals

The physical data of captured small mammals were measured and recorded for morphological species identification [49, 65], including fur color, head and body length, tail length, ear length, hind foot length, gender, and body weight. Species age groups were classified based on body weight data: *A. agrarius* [67], juvenile group (≤ 16.0 g), sub-adult group (16.1–23.0 g), adult group (23.1–37.0 g), and elderly group (> 37.0 g); *R. losea* [67], juvenile group (≤ 37.0 g), sub-adult group (37.1–65.0 g), adult group (65.1–92.0 g), and elderly group (> 92.0 g); *R. norvegicus* [1], juvenile group (≤ 60.0 g), sub-adult group (60.1–110.0 g), adult group (110.1–160.0 g), and elderly group (> 160.0 g).

On the basis of morphological species identification, DNA barcoding molecular biology technology was then used for auxiliary identification. The details are as follows: muscle tissue of the ear margin was cut, DNA was extracted using a genomic DNA Extraction Kit (DP304, Tiangen, Beijing, China), according to the manufacturer’s instructions, and amplification of the *Cytb* (Cytochrome b) [29, 33] and *Cox1* (Cytochrome c oxidase subunit I) [46] genes was conducted using two pairs of universal primers, respectively. The PCR reaction system was as follows: 1 μL of DNA template, 12.5 μL of Premix Taq (Takara, Beijing, China), 1 μL of forward and reverse primers (Sangon Biotech, Shanghai, China), and 9.5 μL of RNase-free water (Takara). All PCR products were sent to Sangon Biotech Co., Ltd for agarose gel electrophoresis and sequencing. Information on PCR primers is shown in Table 1. The annealing temperature and running time were in accordance with previously published data.

**Table 1:** General information on PCR primers used in the study.

Species	Name	Genetic marker	Primer Sequence (5′-3′)	Length (bp)	Reference
Small mammals	*BatL5310*	*Cox1*	CCTACTCRGCCATTTTACCTATG	750	[46]
	*R6036R*		ATCTCTGGGTGTCCAAAGAATCA		
	*L14724*	*Cytb*	CGAAGCTTGATATGAAAAACCATCGTT	1140	[29, 33]
	*H15915*		AACTGCAGTCATCTCCGGTTTACAAGA		
*S. japonicum*	*Sjr2F*	*Sjr2*	GAGGAAACCGAAAGGCACCTA	176	[15]
	*Sjr2R*		CAGCGTTGGGTTGATTTCG		

### Detection of *Schistosoma japonicum* in small mammals

The liver is an important tissue for *S. japonicum* development and egg storage. All small mammal samples were dissected, and liver tissues were separated and stored in 25.0 mL sterile tubes (BS-25-M-S, Biosharp, Beijing, China) individually with 95% ethanol and stored at −80 °C. The experimental steps were as follows: approximately 200 mg of the frozen liver was randomly cut, placed in a 2.0 mL centrifuge tube, 200 μL of ultrapure water was added, a hand-held tissue grinder (MY-20, Jinxin, Shanghai, China) was used to grind the sample into a suspension, and a genomic DNA Extraction Kit (Tiangen) was used to extract DNA, according to the manufacturer’s instructions. The *Sjr2* (non-long terminal repeat retrotransposon) gene of *S. japonicum* was amplified with specific primers [15]. The PCR reaction system was as follows: 1 μL template DNA, 12.5 μL DNA polymerase, 1 μL forward and reverse primers, and 9.5 μL RNase-free water (Takara). A negative control (dH_2_O) group was set for each reaction. All PCR products were sent to Sangon Biotech Co., Ltd for agarose gel electrophoresis and sequencing. BLAST (http://www.ncbi.nlm.nih.gov/BLAST) was then used for sequence alignment to identify small mammals infected with *S. japonicum*. The sequence of PCR primers is shown in Table 1, and the annealing temperature and running time were in accordance with previously published data.

### Statistical analysis

First, the number of traps set up, collected, and small mammals captured were counted. A small number of traps may have been accidentally triggered by other large and medium-sized animals (e.g. wild boars, cattle) in the lake area. Thus, we defined these traps as invalid. The number of valid traps was defined as the number of collected traps – the number of invalid traps. The capture rate of small mammals was defined as the number of small mammals captured / the number of valid traps × 100%.

The One-sample Kolmogorov–Smirnov test was used to test the normality of the data. The Mann–Whitney U test was then used to compare the difference between the number of traps set up and the number of valid traps on lakeside farmland and beaches. The differences in quantitative composition of the small mammal species captured in different seasons and different sampling sites were also evaluated using Mann–Whitney U test. The chi-square test was used to compare the differences in the capture rate of small mammals and the prevalence of *S. japonicum* between different seasons, sites, and habitats. All statistics were calculated in SPSS 22.0 (version 22.0).

Finally, the small mammal *Cox1* and *Cytb* sequences obtained by sequencing were transformed into FASTA format. Mega 11 [50] was used for DNA sequence alignment and adjustment. DnaSP (version 6) [47] was used for nucleotide diversity and haplotype analysis. A DNA sequence substitution saturation test was performed in DAMBE (version 5) [58], and the unsaturated data of base substitution were used to construct the phylogenetic tree. The optimal nucleotide substitution model was calculated using Jmodeltest (version 2.1.10) [13]. Following calculation, the optimal nucleotide substitution models of *Cox1* and *Cytb* sequences were HKY + G and GTR + I + G, respectively. Mega (version 11) was used to construct the Maximum Likelihood (ML) tree, which was iterated 1,000 times. Figtree (version 1.4.4) was used to edit and refine the tree [45].

The representative gene sequences have been deposited in GenBank database under accession numbers PZ428922–PZ428923, PZ428927–PZ428929, PZ766240–PZ766260, and PZ789091–PZ789116.

## Results

We originally planned to carry out four consecutive quarterly sampling sessions (in September 2021, December 2021, March 2022 and June 2022) at four sampling locations. However, sampling personnel were unable to collect samples in March 2022 due to the impact of the COVID-19 pandemic at that time. Therefore, during implementation of the project, only three quarters of small mammal sampling was carried out.

### Information on snap traps and captured small mammals

During the survey, a total of 6,282 (299.14 ± 19.78) traps were set up, 6,123 (291.57 ± 23.61) were collected, 185 (8.80 ± 5.57) were invalid, and a total of 5,938 (282.76 ± 23.84) were valid, including 3,173 in lakeside farmland and 2,765 on beaches (Table 2). There was no significant difference in the number of traps set up (*Z* = −1.113, *p* = 0.265) and the number of valid traps (*Z* = −1.234, *p* = 0.217) between lakeside farmland and beach habitats.

**Table 2:** Statistics on the number of snap traps set up and collected, and small mammals captured.

Sampling season	Sampling site	Lakeside Farmland					Beach				
		No. traps set up	No. traps collected	No. invalid traps	No. valid traps	No. captured animals (%)	No. traps set up	No. traps collected	No. invalid traps	No. valid traps	No. captured animals (%)
2021.09	LH	316	311	15	296	58 (19.59)	226	205	7	198	0 (0)
	HH	322	318	20	298	20 (6.71)	304	298	8	290	6 (2.07)
	NJS	320	316	7	309	24 (7.77)	320	307	5	302	4 (1.32)
	ZX	300	295	3	292	16 (5.48)	292	287	9	278	8 (2.88)
2021.12	LH	295	292	6	286	84 (29.37)	300	295	1	294	8 (2.72)
	HH	300	297	4	293	21 (7.17)	300	296	8	288	3 (1.04)
	NJS	299	297	4	293	3 (1.02)	297	292	1	291	0 (0)
	ZX	299	295	8	287	41 (14.29)	300	297	7	290	11 (3.79)
2022.06	LH	300	264	17	247	22 (8.91)	278	270	13	257	9 (3.50)
	HH	315	309	18	291	7 (2.41)	300	293	16	277	26 (9.39)
	NJS^*^	**–**	**–**	**–**	**–**	**–**	**–**	**–**	**–**	**–**	**–**
	ZX^#^	299	289	8	281	38 (13.52)	**–**	**–**	**–**	**–**	**–**
	Total	3365	3283	110	3173	334 (10.53)	2917	2840	75	2765	75 (2.71)

^*^A flood inundated the road to Nanjishan Township in summer.

^#^The beaches were flooded by water in summer.

There was no significant difference in capture rate between sampling seasons: 2021.09 *vs.* 2021.12 *vs*. 2022.06, 136/2263 *vs.* 171/2322 *vs*. 102/1353, *χ*^2^ = 4.437, *p* = 0.109; the capture rate was significantly different between sampling sites: LH *vs*. HH *vs*. NJS *vs.* ZX, 181/1578 *vs.* 83/1737 *vs.* 31/1195, 114/1428, *χ*^2^ = 100.74, *p* < 0.001; the capture rate was significantly different between habitats: beaches *vs.* lakeside farmland, 334/3173 *vs.* 75/2765, *χ*^2^ = 140.658, *p* < 0.001.

A total of 409 rodents were captured, with a total capture rate of 6.89% (409/5938), of which the total capture rate in the lakeside farmland habitat was 10.53%, and the highest capture rate of lakeside farmland was in LH Township in December 2021, at 29.37% (84/286) (Table 2). The total capture rate in the beach habitat was 2.71%, and the highest capture rate in the beach habitat in HH farm in June 2022 was 9.39% (26/277) (Table 2). The *χ*^2^ test showed that the total capture rate of small mammals was significantly different between lakeside farmland and beach habitats (*χ*^2^ = 140.658, *p* < 0.001), as well as between sites (*χ*^2^ = 100.74, *p* < 0.001) (Table 2). However, there was no significant difference between seasons (*χ*^2^ = 4.437, *p* = 0.109).

### Species composition of small mammal communities identified by morphology

After morphological identification, 409 captured small mammals belonged to three species of Rodentia and one species of Eulipotyphla, which were *Apodemus agrarius*, *Rattus losea*, *R. norvegicus*, and *Crocidura attenuata*, respectively. Among them, *A. agrarius* was the dominant species (72.12%, 295/409) in the small mammal community in the Poyang Lake region, with 239 and 56 captured in lakeside farmland and beaches, respectively (Table 3). This was followed by 106 (25.92%) *R. losea*; 89 and 17 were captured in farmlands and beaches, respectively (Table 3). Only 3 *R. norvegicus* (0.73%) were captured, including 2 in farmland and 1 on beaches (Table 3). Five *C. attenuata* (1.22%) were captured, including 4 in farmland and 1 on beaches (Table 3). From the quantitative composition of the four small mammal species captured, there were no significant differences among the sampling habitats (*Z* = −1.112, *p* = 0.266, Table 3), seasons (Table 4) and sites (Table 5).

**Table 3:** Species composition of the small mammal community in lakeside farmland (F) and beaches (B) in the Poyang Lake region.

Sampling season	Species (No.)	LH		HH		NJS		ZX		Total	
		F	B	F	B	F	B	F	B	F	B
2021.09	*A. agrarius* (101)	44	0	15	6	21	3	7	5	87	14
	*R. losea* (29)	14	0	5	0	2	1	5	2	26	3
	*R. norvegicus* (2)	0	0	0	0	1	0	1	0	2	0
	*C. attenuata* (4)	0	0	0	0	0	0	3	1	3	1
2021.12	*A. agrarius* (148)	79	8	19	2	2	0	35	3	135	13
	*R. losea* (22)	4		2	1	1	0	6	8	13	9
	*R. norvegicus* (0)	0	0	0	0	0	0	0	0	0	0
	*C. attenuata* (1)	1	0	0	0	0	0	0	0	1	0
2022.06*	*A. agrarius* (46)	7	5	6	24	–	–	4	–	17	29
	*R. losea* (55)	15	3	1	2	–	–	34	–	50	5
	*R. norvegicus* (1)	0	1	0	0	–	–	0	–	0	1
	*C. attenuata* (0)	0	0	0	0	–	–	0	–	0	0
Sub-total	*A. agrarius* (295)	130	13	40	32	23	3	46	8	239	56
	*R. losea* (106)	33	3	8	3	3	1	45	10	89	17
	*R. norvegicus* (3)	0	1	0	0	1	0	1	0	2	1
	*C. attenuata* (5)	1	0	0	0	0	0	3	1	4	1
Total		164	17	48	35	27	4	95	19	334	75

*In summer, a flood inundated the road to Nanjishan Township, and the beaches in Zhouxi Township were flooded by water.

**Table 4:** Statistical values of the *t-*test were used to compare the difference in species composition of small mammals between sampling seasons.

	2021.09	2021.12	2022.06
2021.09		***Z* = −0.577, *p* = 0.564**	***Z* = −0.8**71**, *p* = 0.3**84
2021.12	*Z* = −0.296, *p* = 0.767		***Z* = −0.2**96**, *p* = 0.7**57
2022.06	*Z* = −0.292, *p* = 0.770	*Z* = −0.296, *p* = 0.767	

Bold font, upper-right, represents the data in lakeside farmland; grey font, lower-left, represents the data on beaches.

**Table 5:** Statistical values of the *t-*test were used to compare the difference in species composition of small mammals between sampling sites.

	LH	HH	NJS	ZX
LH		***Z* = −0.592, *p* = 0.686**	***Z* = −0.584, *p* = 0.559**	***Z* = −436, *p* = 0.663**
HH	*Z* = **−**0.149, *p* = 0.882		***–***	***Z* = −1.162, *p* = 0.245**
NJS	*Z* = **−**0.899, *p* = 0.369	*Z* = **−**0.464, *p* = 0.642		***Z* = −1.169, *p* = 0.243**
ZX	*–*	*Z* = **−**0.296, *p* = 0.767	*Z* = **−**1.042, *p* = 0.343	

Bold font, upper-right, represents the data in lakeside farmland; grey font, lower-left, represents the data on beaches.

### Species composition of small mammals identified by mitochondrial DNA

**Figure 2:**
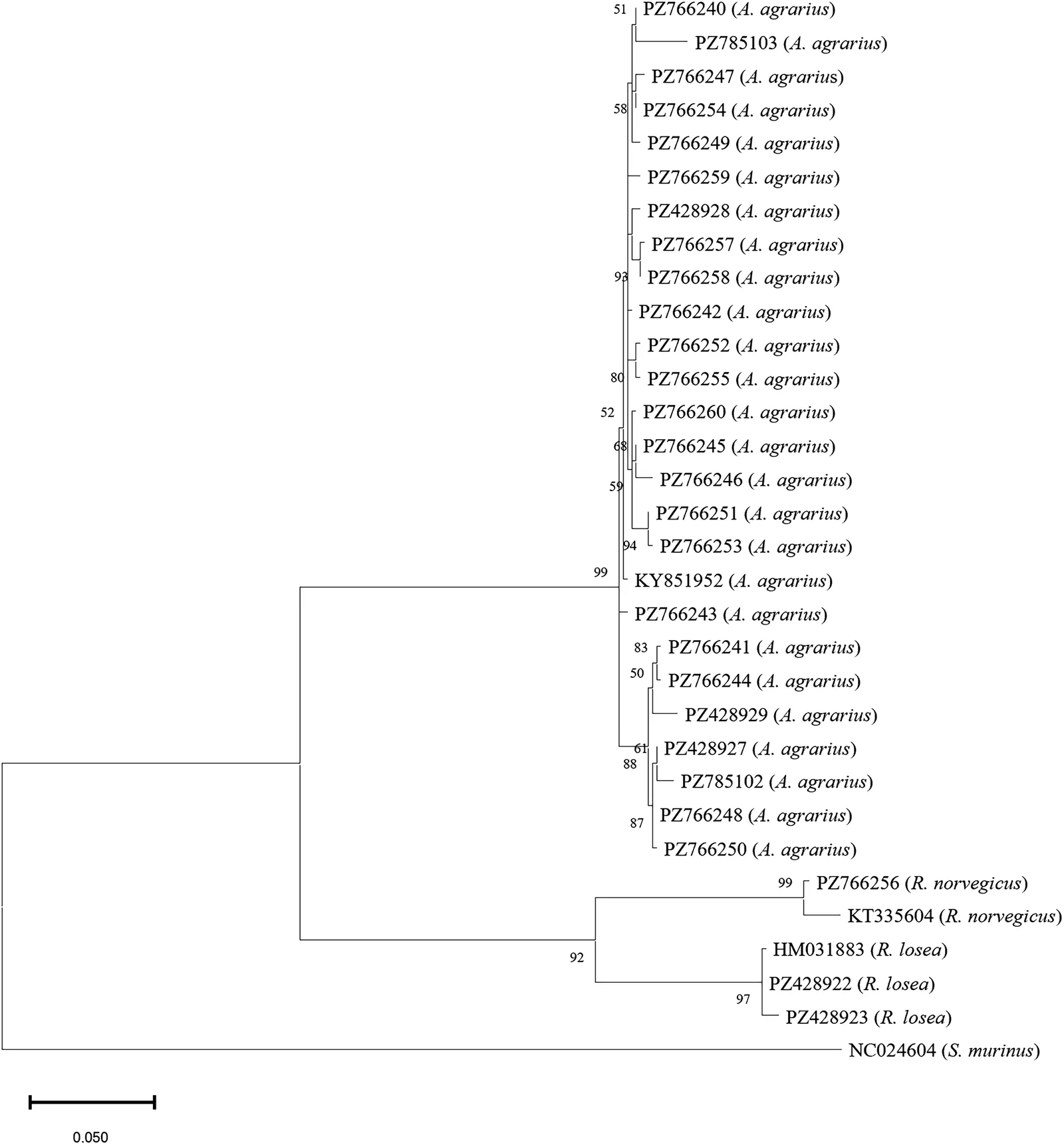
Phylogenetic analyses of the Cytochrome c oxidase subunit I (*Cox1*) gene of small mammals with the maximum likelihood method.

**Figure 3:**
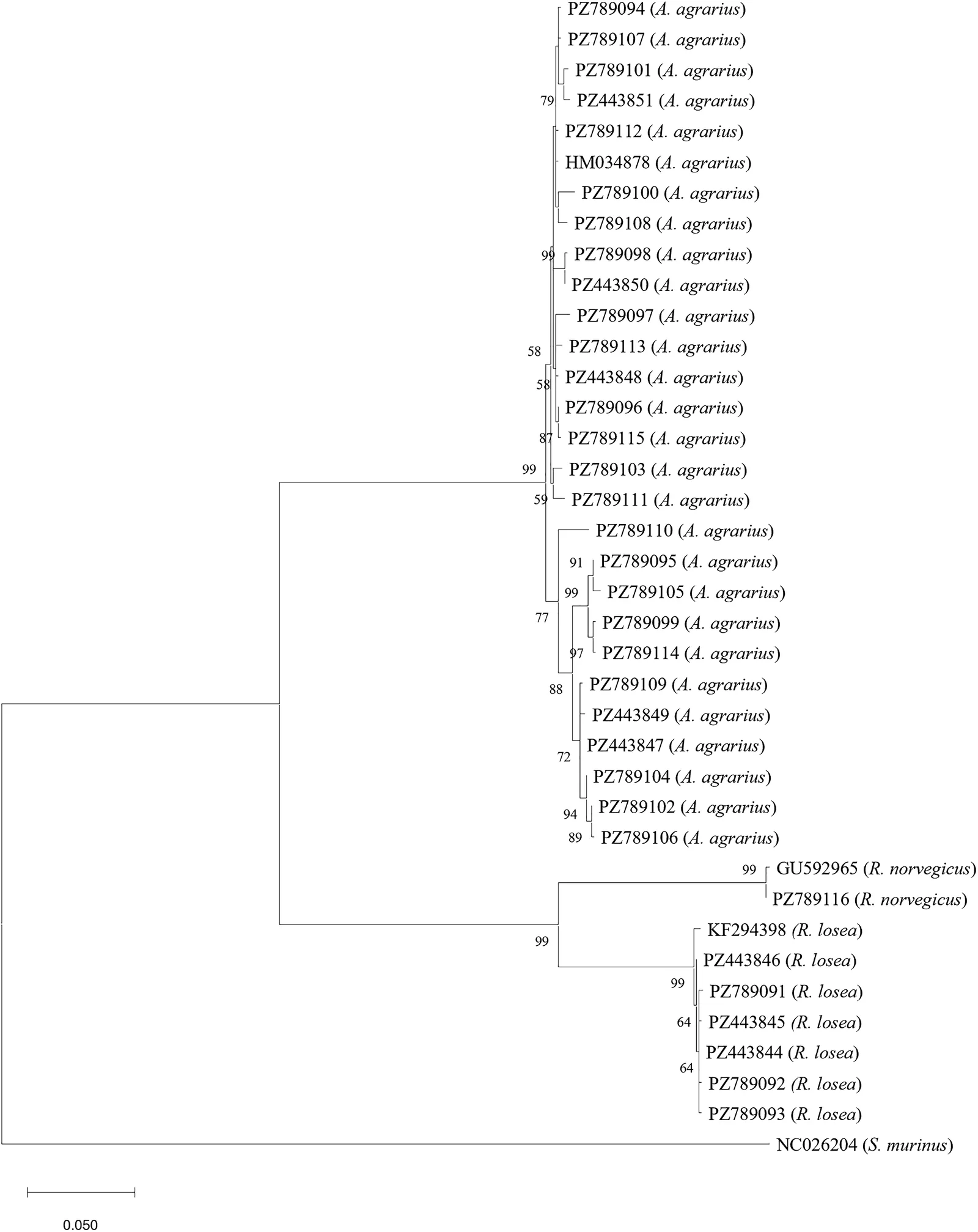
Phylogenetic analyses of the Cytochrome b (*Cytb*) gene of small mammals with the maximum likelihood method.

In this study, a total of 167 captured small mammal sequences were analyzed by phylogenetic tree construction, including 70 samples from LH (*A. agrarius n* = 48, *R. losea n* = 22), 29 samples from HH (*A. agrarius n* = 22, *R. losea n* = 7), 49 samples from ZX (*A. agrarius n* = 13, *R. losea n* = 36), and 19 samples from NJS (*A. agrarius n* = 14, *R. losea n* = 4, *R. norvegicus n* = 1). The *Cox1* and *Cytb* sequences were obtained. After DnaSP analysis, 28 haplotypes of the *Cox1* sequence and 34 haplotypes of the *Cytb* sequence were obtained. At the same time, the sequences of *Suncus murinus* (NC024604, as the outgroup), *A. agrarius* (KY851952, HM034878), *R. losea* (HM031883, KF294398), and *R. norvegicus* (KT335604, GU592965) were downloaded from NCBI. The phylogenetic tree was constructed for molecular species identification, and the results were compared with those of morphological identification. The topological structure of the ML tree of small mammals in Poyang Lake region based on *Cox1* (Fig. 2) and *Cytb* (Fig. 3) was similar. *Apodemus agrarius*, *R. losea*, and *R. norvegicus* clustered on their respective branches, and the identification results were consistent with those based on morphology. Both *Cox1* and *Cytb* primers effectively identified small mammals in the Poyang Lake region.

### Prevalence of *Schistosoma japonicum* in small mammals

In this study, 394 liver DNA samples from small mammals were successfully extracted, and a total of 11 positive samples were detected (2.79%, 11/394). Of these, 8 samples were *A. agrarius*; the total prevalence of *A. agrarius* was (2.85%, 8/281). In all, 3 samples were *R. losea*; the total prevalence of *R. losea* was (2.83%, 3/106). No positive samples were detected in *R. norvegicus* (0, 0/3) and *C. attenuata* (0, 0/4) (Table 6). The chi-square test showed that the differences in species, gender, age, sampling location, sampling season and sampling habitat were not significant factors influencing the prevalence of *S. japonicum* in small mammals (Table 6).

**Table 6:** Prevalence of *Schistosoma japonicum* in the small mammal community of the Poyang Lake region.

Category		No. examined	No. positive (%, 95% CI)	Statistical test
Host Species	*A. agrarius*	281	8 (2.85, 1.35–5.61)	*χ* ^2^ = 0.205, *p* = 0.977
	*R. losea*	106	3 (2.83, 0.61–0.83)	
	*R. norvegicus*	3	0 (0)	
	*C. attenuata*	4	0 (0)	
Sex^*^	Male	201	6 (2.99, 1.23–6.54)	*χ* ^2^ = 0.022, *p* = 0.564
	Female	183	5 (2.73, 1.00–6.41)	
Age^#^	Juvenile	39	1 (2.56, 0.00–14.36)	*χ* ^2^ = 3.992, *p* = 0.262
	Sub-adult	117	5 (4.27, 1.58–9.87)	
	Adult	133	5 (3.76,1.45–8.04)	
	Elderly	94	0 (0)	
Sampling season	2021.09	133	1 (0.75, 0.00–4.56)	*χ* ^2^ = 5.159, *p* = 0.076
	2021.12	160	8 (5.00, 2.40–9.71)	
	2022.06	101	2 (1.98, 0.11–7.37)	
Sampling site	LH	174	5 (2.87, 1.05–6.73)	*χ* ^2^ = 3.623, *p* = 0.305
	HH	81	0 (0)	
	NJ	29	1 (3.45, 0.00–18.63)	
	ZX	110	5 (4.55, 1.69–10.47)	
Habitat	Farmland	320	10 (3.12, 1.63–5.73)	*χ* ^2^ = 0.404, *P* = 0.697
	Beach	74	1 (1.35. 0.00–7.97)	
Total		394	11 (2.79, 1.51–4.99)	

^*^The sex of 10 individuals could not be identified, including 3 *A. agrarius*, 4 *R. losea*, and 3 *C. attenuate*.

^#^Due to morphological damage, the age group of 11 individuals could not be classified, including 3 *A. agrarius,* 4 *R. losea,* and 4 *C. attenuate*.

## Discussion

Surveillance of *S. japonicum* prevalence in small mammals has been included in the national action plan for schistosomiasis elimination [60, 66]. Poyang Lake region has always been a key area for schistosomiasis japonica control in China. In recent years, with the implementation of policies aimed at closing the lake to grazing and fishing, the reduction in human disturbance, and improvement in the ecological environment, an increase in the number of wild small mammals in the lake area is expected. A thorough understanding of the ecological and epidemiologic factors affecting wild small mammal populations is important for the prevention and control of schistosomiasis japonica.

### High relative density of small mammals

The method of snap-trapping can be used for species identification and disease detection in individual captured animals, as well as to easily and effectively estimate the relative density of small animals in the whole region based on the capture rate [65]. There was no significant difference in the relative density of small mammals between different seasons (Table 2). However, there was a significant difference in the density between different sampling sites. The lowest density was only 2.59% in NJS, but the highest density was 11.47% in LH (Table 2). This result suggests that it is important to increase the number of sampling sites at a certain sampling frequency, while carrying out a community survey of small mammals in the Poyang Lake region.

The national rodent monitoring survey reported that the relative density of farmland rodents in Jiangxi Province was 2.10% [42]. However, the monitoring data in the present study showed that the density in lakeside farmland was as high as 10.53%, and the density on beaches was 2.71% (Table 1), which were higher than the density of farmland rodents in the whole province. Similarly, the rodent density in lakeside farmland of the Poyang Lake region from 1997 to 2002 was 19.73% (1308/7115) [10], while survey results from 2015 to 2016 showed that the density of small mammals in lakeside farmland was 10.12% (375/3706), and that on beaches, it was 5.47% (155/2832) [9]. In general, the small mammals in the Poyang Lake region may have a high population density, and numbers in the lakeside farmland habitat were significantly higher than in beach habitats.

Additionally, in Dongting Lake, the second largest freshwater lake in China, located in the middle and lower reaches of the Yangtze River in Hunan Province, the density distribution of rodents was completely different to that in Poyang Lake. Monitoring that lasted for more than a decade (2003–2018) indicated that the capture rate of small mammals on the beach (9.51%, 2,551/26,821) was significantly higher than that in lakeside farmland [17, 64]. However, further comparison and analysis of this inconsistent finding requires more long-term data for the Poyang Lake region.

### Wild small mammal community composition

Due to the suitable ecological environment, the Poyang Lake region is rich in small mammals. According to relevant literature data, approximately 16 species of small mammals may be distributed in the area [49, 51, 65]. In this study, only four small mammal species, *A. agrarius*, *R. losea*, *R. norvegicus*, and *C. attenuata*, were recorded. *Apodemus agrarius* and *R. losea* have always been the dominant species here [11]. Due to limited logistic support, only four sampling sites in lakeside farmland and beach habitats were selected for small mammal collection. No significant difference in the numbers of the four small mammal species was observed between different sampling sites (Table 4) and seasons (Table 5), showing that these species are widely distributed in the Poyang Lake region in different seasons and locations. In future surveys of small mammals in local lake regions, it is suggested that the number of sampling counties and the type of habitats (such as islands, woodlands) should be increased to obtain more information on the diversity of small mammal species. This would provide more comprehensive background data supporting schistosomiasis japonica monitoring and control in wild small mammals.

Species classification and identification are an important basis for research on the monitoring of pathogens and disease, as well as species management and protection. DNA barcoding has become an important technology for mammalian species classification and identification [30, 31], with the advantages of being unaffected by individual development stage, and damage or deformity of morphological characteristics [28]. In this survey, morphological identification and DNA barcoding technology based on the *Cox1* (Fig. 2) and *Cytb* (Fig. 3) genes effectively distinguished these small mammal species distributed in the Poyang Lake region. However, with the sole use of traditional morphological classification methods, certain species of small mammals may not be detected [6, 20]. Therefore, in future research of the small mammal community in the Poyang Lake region and in Jiangxi Province, it is recommended that classical morphology continue to be combined with molecular biology to identify species composition and increase the reliability of results.

### Prevalence of *Schistosoma japonicum* and the importance for schistosomiasis control

The prevalence of *Schistosoma* spp. in wild small mammals is a concern for researchers worldwide. Prevalence was reported to be 1.9%–28.6% in the area around the Senegal River Basin in West Africa [4], and 6.0% in another survey in western Kenya [24]. Relevant studies have shown that the molecular prevalence of *S. japonicum* in small mammals was significantly higher than that in buffalo [18]. In fact, buffalo were considered to be one of the main hosts and sources of *S. japonicum* infection in China. The application of DNA-PCR technology provides a more convenient and sensitive molecular detection method. In a rabbit model, the target DNA fragment was amplified by PCR from liver homogenate, eggs purified from liver, fecal eggs, and sera [37, 57]. In the Poyang Lake region, the total prevalence of *S. japonicum* in the local small mammal community was 2.79% (11/394). According to research data from other provinces located in the middle and lower reaches of the Yangtze River, the prevalence of *S. japonicum* in *R. norvegicus* was 59.8% (113/198) on two beaches in Nanjing, Jiangsu Province in 1996 [59], and prevalence *Rattus* spp. reached 10.62% (17/168) on five islands in the Dongting Lake region, Hunan, in 2011 [23]. Compared with these data, the overall prevalence of *S. japonicum* in the Poyang Lake region is at a relatively low level, which reflects the constant efforts of Jiangxi Province over the past 70 years. The implementation of comprehensive prevention and control strategies, such as replacing cattle with machines, closing the island to prohibit grazing, and keeping livestock in captivity, has achieved remarkable results. At present, the infection rate of *S. japonicum* in humans and domestic animals is at a very low level [26, 35].

However, the prevention and control of schistosomiasis japonica in the middle and lower reaches of the Yangtze River in China still faces a series of challenges [61]. For example, some areas have not controlled schistosomiasis transmission [62], and there is still a resurgence of schistosomiasis in areas where it was completely eliminated [32]. In Hubei province, the prevalence of *S. japonicum* in small mammals such as *R. norvegicus*, *R. losea*, and *R. flavipectus* was between 10.77% and 19.18% [63]. Data monitoring in Anhui Province showed that the prevalence of *S. japonicum* in *A. agrarius* and *R. flavipectus* in some endemic villages could be as high as 54.76% (23/42) and 62.64% (21/33), respectively, in recent years [53]. In addition, *R. norvegicus* and *R. flavipectus* were also the main small mammal species infected with *S. japonicum* [8, 16, 25]. In Jiangxi Province [38], the infection rate of *S. japonicum* in small mammals in mountainous areas was 2.91% (5/172), of which *R. norvegicus* and *R. flavipectus* were the main infected species. Combined with the results of our study (Table 6), in the middle and lower reaches of the Yangtze River, China, *R. norvegicus*, *A. agrarius*, and *R. flavipectus* may be the most important wild small mammal hosts of *S. japonicum*. The number of *R. norvegicus* captured in the Poyang Lake region was limited (3/409), which may be the reason for no *Sjr2* sequence being detected in this species. *Crocidura attenuata* was also considered one of the definitive hosts of *S. japonicum* [27], but no positive samples were detected in this study. The epidemic information on *C. attenuata* in the middle and lower reaches of the Yangtze River requires further monitoring.

For the population of *R. norvegicus*, a survey showed that the infection risk of *S. japonicum* was significantly higher in larger and older individuals [59]. In a small mammal community, the infection rate of *S. japonicum* would be significantly different in different species [8, 53]. However, based on our data, there was no significant difference in the prevalence of *S. japonicum* between species of different ages and other factors including different sampling sites, habitats, seasons, and host gender (Table 6). As the main wild definitive hosts in the Poyang Lake region, the prevalence and dynamic trend of schistosomiasis japonica in *A. agrarius* and *R. losea* populations are important factors in the future elimination of schistosomiasis in the area. Finally, in this study, we used only the PCR method to detect *S. japonicum* DNA in the liver of small mammals. When infection intensity is low or there is single-sex infection, the limitations of this DNA-PCR method may account for the inability to detect a positive signal. There continues to be a need for efficient and accurate detection technology for schistosomiasis infection in wild small mammals for future schistosomiasis elimination.

## Conclusion

Determining the small mammal community composition and prevalence of *Schistosoma japonicum* is the basis for effective prevention and control. In this survey, four small mammals, *Apodemus agrarius, Rattus losea*, *R. norvegicus*, and *Crocidura attenuata*, were captured and identified in the Poyang Lake region, which is a typical endemic area of schistosomiasis japonica. *Apodemus agrarius* and *R. losea* were the dominant species and the main species infected with *S. japonicum,* with a total prevalence of 2.79%. Our findings revealed the molecular prevalence of *S. japonicum* in the wild small mammal community in the Poyang Lake region for the first time and provide basic data for the transmission and control of *S. japonicum* in these animals. Therefore, it is suggested that continuous monitoring of the small mammal community should be carried out in future schistosomiasis prevention and control programs. Increasing the number of sampling sites, analyzing the risk of wild small mammals in the transmission of schistosomiasis under the condition of low prevalence, and paying more attention to the dynamic changes in schistosomiasis prevalence in *A. agrarius* and *R. losea* are approaches that may contribute to the elimination of schistosomiasis in the Poyang Lake region of Jiangxi Province and in other regions of China.
